# An Analysis of the Performance and Comfort Properties of Fire-Protective Material by Using Inherently Fire-Retardant Fibers and Knitting Structures

**DOI:** 10.3390/ma16237347

**Published:** 2023-11-25

**Authors:** Awais Ahmad Khan, Hafsa Jamshaid, Rajesh Kumar Mishra, Vijay Chandan, Viktor Kolář, Petr Jirků, Miroslav Müller, Shabnam Nazari, Tatiana Alexiou Ivanova, Tanveer Hussain

**Affiliations:** 1 School of Engineering and Technology, National Textile University, Sheikhupura Road, Faisalabad 37610, Pakistan; askhan181199@gmail.com (A.A.K.); hafsa@ntu.edu.pk (H.J.); hussain.tanveer@gmail.com (T.H.); 2 Department of Material Science and Manufacturing Technology, Faculty of Engineering, Czech University of Life Sciences Prague, Kamycka 129, Suchdol, 165 00 Prague, Czech Republic; vijay@tf.czu.cz (V.C.); vkolar@tf.czu.cz (V.K.); jirkup@tf.czu.cz (P.J.); muller@tf.czu.cz (M.M.); 3 Department of Sustainable Technologies, Faculty of Tropical AgriSciences, Czech University of Life Sciences Prague, Kamycka 129, Suchdol, 165 00 Prague, Czech Republic; nazari@ftz.czu.cz (S.N.); ivanova@ftz.czu.cz (T.A.I.)

**Keywords:** fire-resistant (FR) material, knitted structure, inherently fire-retardant fiber, station suits, mechanical performance, comfort properties

## Abstract

This paper investigates the development of fabric materials using several blends of inherently fire-resistant (FR) fibers and various knitted structures. The samples are evaluated with respect to their performance and comfort-related properties. Inherently fire-resistant fibers, e.g., Nomex, Protex, carbon and FR viscose, were used to develop different structures of knitted fabrics. Cross-miss, cross-relief, and vertical tubular structures were knitted by using optimum fiber blend proportions and combinations of stitches. Several important aspects of the fabric samples were investigated, e.g., their physical, mechanical and serviceability performance. Thermo-physiological and tactile/touch-related comfort properties were evaluated in addition to flame resistance performance. An analysis of mechanical performance indicated that the knitted structure has a significant influence on the tensile strength, bursting strength and pilling resistance. The cross-relief structure proved to be the strongest followed by the cross-miss and vertical tubular structures. The FR station suits made from 70:30 Protex/Nomex exhibited the best combination of tensile and bursting strength; therefore, this material is recommended for making a stable and durable station suit. Interestingly, it was also concluded from the experimental study that knitted samples with a cross-relief structure exhibit the best fire-resistance performance. Fiber blends of 70:30 Protex/Nomex and 70:30 Nomex/carbon were found to be optimum in terms of overall performance. The best flame resistance was achieved with Nomex:carbon fiber blends. These results were confirmed with vertical flammability tests, TGA, DTGA and cone calorimetry analysis. The optimization of blend composition as well as knitting structure/architecture is a crucial finding toward designing the best FR station suit in terms of mechanical, dimensional, thermal, thermo-physiological and flame resistance performance.

## 1. Introduction

Fire hazards are one of the major concerns causing loss of life around the world. Therefore, it is necessary to ensure fire safety in any workplace [1]. Firefighters play a very important role in dealing with fire hazards and saving lives. Fire-resistant fabrics in this regard have a very wide range of applications, e.g., industrial uniforms, automobile upholstery, office materials and most importantly, the turnout gears used by the firefighters. The fire-resistant fabric materials worn underneath the firefighters’ turnout gear are called station suits/garments. Station suits require fire safety along with the optimum comfort of wearing, which can enhance the performance and efficiency of the firefighters/workers. Firefighters’ protective clothing minimizes skin burn and ensures safety during firefighting operations and rescue missions. A recently increasing area of research regards the development of self-powered fire-warning materials that can be incorporated into the firefighters’ clothing to achieve active fire protection before the protective clothing catches fire [2]. This approach is more precision based with high-tech installations of thermoelectric textile (TET)-based fire warning electronics. However, a passive fire protection can be achieved by using inherently fire-resistant fibers and suitable fabric structures [3].

There are various aspects of clothing comfort, e.g., thermo-physiological comfort which includes air permeability, thermal resistance and moisture management of the fabrics. Another subjective factor of comfort is tactile/touching comfort, which defines the sensorial behavior of the fabrics when it comes in contact with the wearer’s body. Tactile comfort includes softness, smoothness, drapability, wrinkle recovery, elasticity, flexibility, surface roughness, thickness and weight [4].

There exists a microclimate between the inner garment and the human body in a clothing system. The fire-resistant fabrics worn next to the skin must have sufficient tactile and thermo-physiological comfort along with enhanced fire safety [5]. The discomfort related to the current fire-resistant fabrics includes poor moisture/vapor management, which accounts for the accumulation of sweat inside the uniform. It can cause severe skin burn injuries at extreme temperatures. The microclimate between the fabric material and the human skin should maintain a balance between the flame resistance and thermo-physiological strain. The use of proper fabric structure and the composition of fiber materials is vital for achieving this purpose [6].

Researchers have investigated the fire resistance of fabric materials used as station suits/uniforms. It was concluded that station suits must have satisfactory comfort properties, mechanical performance and fire resistance. The effect of fire-retardant agents on the acrylic fibers and their flammability and touch-related properties were evaluated. It was concluded that the combustion retardation time for the chemically treated fibers was increased, while there was deterioration in the tactile comfort properties [5,6]. Asbestos and glass fibers were used for flame retardance, but they proved to be harmful and not suitable for use in station suits [7].

In recent times, numerous fibers, e.g., meta-aramid, para-aramid, carbon, modacrylic, FR viscose, etc. have been developed with high flame resistance characteristics. It was also noted that the mentioned fibers lack optimum comfort characteristics except for modacrylic and viscose. The firefighting gear was studied in terms of materials and structural parameters. It was found that the type of fiber plays a very important role in determining thermal resistance. It was also concluded that yarn properties depend on the fiber blend composition and have a major impact on the fire resistance performance of fabric [8]. Apart from fiber characteristics, the fabric structure also plays an important role in determining the comfort properties. It was concluded that knitting structural parameters, e.g., fabric density, thickness, wales/cm, courses/cm, and porosity have a dominant effect on the thermo-physiological comfort properties [9].

The surface temperature distribution for different kinds of knitted fabrics including cross-tuck, double cross-tuck, cross-miss, rib, interlock and plain structures was studied using a hot plate and an infrared thermal camera. It was concluded that interlock and rib knitted fabrics have better thermal resistance than plain jersey fabrics. The fabrics having miss stitches show higher thermal transmission [10]. Interlock fabrics are dimensionally more stable and are softer than rib and plain single jersey fabrics. It was also concluded that interlock fabrics show higher thermal resistance, which is an important factor for designing the fire safety materials [11]. These fabrics show better diffusion ability, which tends to enhance the overall moisture management capacity (OMMC) of the material. These properties account for the suitability of interlock fabrics in applications next to the skin in the safety uniforms [12].

The flame retardance performance of firefighters’ clothing material has been a focus of research for several decades. As per the available literature, it is still difficult to achieve the optimum level of mechanical, dimensional stability and fire-safety performance along with enhanced comfort properties of knitted fabrics while using blends of fire-resistant fibers. The overall mechanical performance (including tensile strength, bursting strength and pilling resistance) is very important to determine the service life of station suits. Therefore, in the present study, evaluation of the relevant mechanical performance is carried out using experimental analysis.

The aim of the present study is to improve the comfort of station suit materials without compromising with the mechanical properties and flame protection performance. Inherently fire-resistant fibers, e.g., Nomex, Protex, carbon and FR viscose, were used to develop different types of knitted materials using optimum fiber blend proportions and combinations of stitches. Nomex fiber was selected as a common component in all the samples, since it is well established in the market as a fire-retardant material [13,14]. The results obtained can be used to engineer FR materials with optimum fire protection and comfort performance.

Optimization of the station suit in terms of the mechanical performance, shape stability, thermal resistance, thermo-physiological comfort as well as flame resistance is a complex process. Statistical tools are used to calculate the errors and standard deviation of selected characteristics. The current research has achieved the objective of this multi-criteria decision making through empirical and experimental results. Further research is in progress for a deeper statistical validation and optimization using the current experimental data.

## 2. Materials and Methods

### 2.1. Materials

Four types of inherently fire-resistant fibers, e.g., Nomex, Protex, carbon and FR viscose, were chosen for this study. Meta-aramid (Nomex) fibers were produced by Dupont (Wilmington, DE, USA), Modacrylic (Protex) fibers were produced by Kanecaron (Hyogo, Japan), polyacrylonitrile (PAN)-based carbon fibers were produced by Zoltek (Totowa, NJ, USA) and FR viscose fibers were produced by Aramid Hpm, LLC (Hilton Head, SC, USA). The properties of fibers are given in Table 1.

### 2.2. Methods

Five different types of yarn samples were prepared with optimum blends of FR fibers to develop the knitted fabric samples. Nomex was selected as a common component in all the fabric samples since it is well known as a fire-retardant material [13,14]. The details of yarn samples are given in Table 2. Ring spun yarns of 20 tex (g/km) were developed at Shahbaz Garments, Faisalabad, Pakistan.

A total of 15 experimental samples of knitted fabrics were prepared by using the yarns composed of FR fiber blends. The knitted fabric materials were produced using three types of interlock knitting structures. Sample specifications were decided based on the previous literature [13,14,15]. It is well known in the literature that the interlock knitted structure and its derivatives are relatively more stable than standard knitted architecture [16,17,18]. For fabrication, we used an Interlock knitting machine from Fukuhara Co., Ltd. (Kobe, Japan) with a diameter of 75 cm and gauge 20 (no. of needles/cm). The designs of the knitted fabric structures were formulated using Wisetex software version 3.2 (developed by Wisetex, Leuven, Belgium) (https://wisetexdemo.software (accessed on 1 September.2023). The simulated designs and specifications of the knitted fabric samples are given in Table 3.

In the interlock knitting machine, the dial and cylinder work together to create the fabric samples. There are two types of needles (short needles and long needles) working together on both the dial and cylinder, respectively. There two types of needles that work conjugately to form an identical appearance on both (front and back) sides of interlock fabrics. Cross-miss is a derivative of the interlock structure which is developed by the combination of knit and miss stitches. The cam profile on the dial and cylinder decides the formation of alternate miss and knit stitches, which results in the formation of cross-miss patterns.

Interlock cross-relief is another derivative structure having subsequent miss stitches on the dial side. On the cylinder side, there is a combination of alternate knit and miss stitches. This results in a comparatively lower number of successive miss stitches and smaller air pockets in the structure.

The interlock vertical tubular structure is a type of architecture that creates a seamless tubular fabric by joining the miss and knit stitches with the help of tuck stitches. The hollow vertical tubes formed between the front and back layers is due to the presence of miss stitches on the dial and cylinder, which are joined with the help of tuck stitches in regular intervals. Such architecture is much more stable and mechanically robust as compared to several other types of flat knitted structures.

Both cross-relief and cross-miss structures have a combination of knit and miss stitches. The cross-relief structure consists of a higher percentage of consecutive miss stitches (approx. 69%). The cross-miss structure consists of alternating miss (approx. 58%) and knit stitches. The vertical tubular structure consists of knit, miss (approx. 50%) and tuck (approx. 8%) stitches together.

All the selected derivatives of interlock architecture are optimum for mechanical performance, dimensional stability as well as porosity, which is essential for the thermo-physiological comfort [14,15,16,17,18]. The combination of stitch and miss stitches results in the stability and balance of stresses in the wales (length wise) and courses (width wise). Thus, the cross-relief and cross-miss architecture result in robust and compact materials suitable for long-term performance against the probable mechanical loads during their use. Furthermore, the knitting geometry in general enables adequate stretch and fit for the wearer. The elasticity of the knitted architecture enables ergonomic comfort and easy body movement. The porous nature of the loops enables optimum heat and mass (moisture) transfer and makes the fabric an ideal choice for functional workwear. Especially for firefighters, these essential performance parameters and efficient fire resistance are of utmost importance. The samples were prepared based on statistical principles and the design of experiment (DOE) as given in Table 4.

### 2.3. Chemical Processing

Chemical processing of all the knitted fabric samples was carried out before their performance evaluation. Scouring and bleaching were completed at pH 11 and a temperature of 600 °C using a standard bleaching agent (non-phosphate-type detergent 2408 ECE) procured from SDC Enterprises, Lahore, Pakistan. A recipe for the bleaching process consisted of hydrogen peroxide, calcium carbonate and a wetting agent. The wet processing was carried out by using a mini jet dyeing machine (SDC Enterprises, Lahore, Pakistan).

### 2.4. Physical Properties of Knitted Samples

The physical properties of knitted fabric samples were evaluated before and after chemical processing. The weight/grams per square meter (GSM) of all fabric samples was measured using the ASTM D 3776 standard method [19]. The thickness of the samples was determined according to the ASTM D1777 [20]. The courses/cm and wales/cm of the samples were counted using a magnifying glass.

### 2.5. Mechanical Properties of Fabric Samples

Tensile tests were carried out using the ASTM D5034-21 standard method on a tensile strength tester (Daiei Kagaku Seiki-KG-300, Kyoto, Japan) [21]. The gauge length of 20 cm and width of 5 cm were used for testing the samples. The crosshead speed was set at 1 cm/min. For each type of sample, 10 measurements were carried out, and the average stress–strain curves were plotted.

The bursting strength of knitted fabrics was determined following the EN ISO 13938-2 standard test method [22]. This test was performed on a bursting tester (Mullen type C, MA, USA) having a diameter of 30 mm using the pneumatic method [22]. The tests were repeated 10 times for each sample, and the average was reported.

The pilling resistance of knitted fabrics was tested in accordance with the ISO 12945-2 standard method [23] on a Martindale tester (Martindale-Hubbell, New Providence, NJ, USA). The pilling was conducted for 5 h, and the grades were determined after 18,000 cycles by comparing with pilling grades/standards. All the tests were performed under standard atmospheric conditions, i.e., temperature 22 ± 2 °C and 65 ± 2% RH.

### 2.6. Dimensional Stability

The dimensional stability of the fabric samples was tested according to ASTM D 2594-99a standard method [24]. The test was carried out using a tensile strength tester (Daiei Kagaku Seiki, KG-300, Kyoto, Japan). The fabric growth, stretch and recovery % were calculated against applied load. The tests were repeated 10 times for each sample, and the average was reported.

### 2.7. Thermo-Physiological Comfort Properties

Air permeability plays a vital role in deciding the comfort of clothing fabric materials. The test was performed on an M021A air permeability tester (SDL ATLAS, South Carolina, USA) in accordance with ASTM-D-737 [25]. Thermal resistance was measured using Alambeta Thermolab (Sensora, Liberec, Czech Republic) according to the ISO-11092 standard [26]. The overall moisture management capacity (OMMC) indicates the management of liquid moisture transport. To investigate this property, an MMT moisture management tester (SDL ATLAS, Rock Hill, SC, USA) was used. The test was performed according to the AATCC-195.OMMC standard method [27]. The test comprises the measurement of wetting time, maximum wetted radius, one-way transport index, absorption rate and spreading speed of the liquid moisture. The tests were repeated 10 times for each sample, and the average was reported.

### 2.8. Tactile/Touching Comfort Properties

Tactile comfort is the subjective evaluation of the human sensation to the fabric materials. The touch/tactile comfort properties were tested using a Phabrometer-3 fabric evaluation system developed by Nu Cybertek, Inc. (Davis, CA, USA) following the AATCC TM 202:2014 standard test method [28]. 

### 2.9. Flame Resistance by Vertical Flammability Test

A vertical flammability test was performed to analyze the burning, melting, and dripping behavior of specimens. The test was performed on a M233B vertical flammability tester (SDL ATLAS, Rock Hill, SC, USA) following the ASTM-D-6413 standard method [29]. All the specimens were prepared in standard size as mentioned in the test method, and the test was conducted under controlled laboratory conditions. The tests were repeated 10 times for each sample, and the average was reported.

### 2.10. Thermogravimetric Analysis

The Mettler Toledo TGA/SDTA851e instrument from (Mettler Toledo, Prague, Czech Republic) was used to study the thermogravimetric behavior (thermal stability and degradation) of the knitted fabric samples. Thermogravimetric analysis was performed under oxidative conditions. The samples were heated from 25 to 700 °C at a heating rate of 10 °C/min to yield the decomposition temperature, weight loss (%), and maximum decomposition peak.

### 2.11. Cone Calorimetry

**Figure 1:**
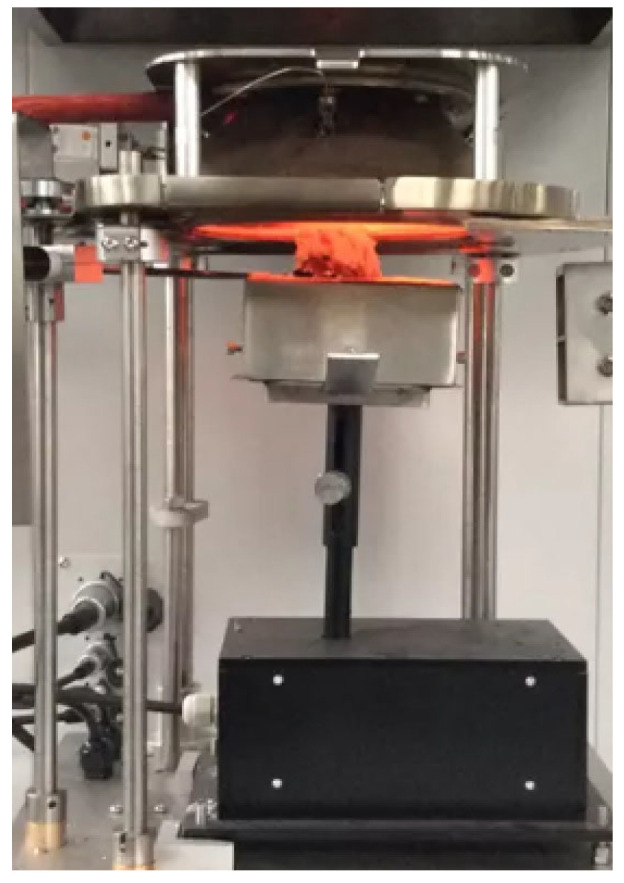
Cone calorimeter test.

An ISO 5660 cone calorimeter (Fire Testing Technology Ltd., East Grinstead, UK) was used to evaluate performance of the fabric samples. This device operates on the ‘oxygen consumption’ principle as per ASTM E 1354/ISO 5660 [30]. A heat flux of 50 kW/m^2^ was used for the samples to determine the heat release rate. The experimental set up is shown in Figure 1.

### 2.12. Steady-State (Convective and Radiant) Heat Resistance

**Figure 2:**
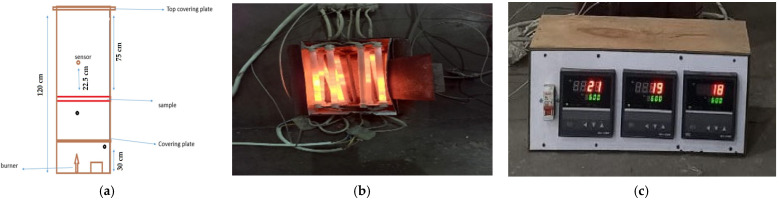
(**a**) Schematic diagram of instrument used for steady-state heat resistance, (**b**) heating rod, (**c**) thermocouple.

This test was conducted to predict the thermal protection performance of fabric materials according to the ASTM F2700-08 standard method [31]. The instrument was equipped with 12 quartz rods for heating purposes. The thermocouple was used to measure the temperature gradient. The schematic diagram of the instrument is shown in Figure 2a. Figure 2b,c show heating rods and thermocouples, respectively.

## 3. Results and Discussion

### 3.1. Physical Properties

**Figure 3:**
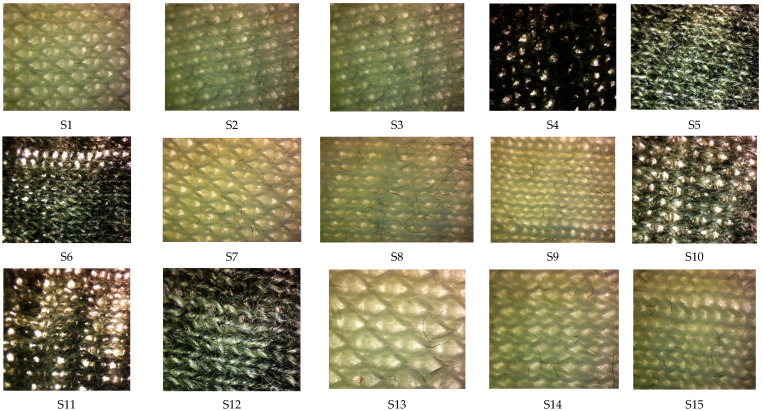
Images of the different knitted fabric samples.

The optical images of all the developed knitted fabric samples as shown in Figure 3 were taken at 1.5 magnification by using an OPTIKA microscope C-B 10 (developed by via Rigla, 30 24010 Ponteranica, Italy).

**Figure 4:**
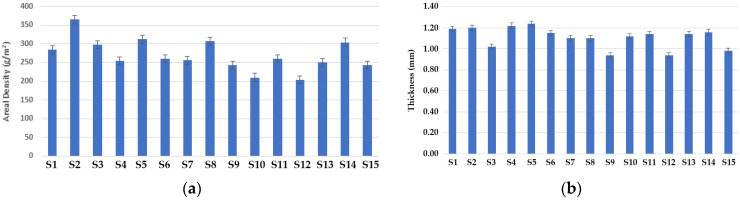
Physical parameters of the knitted fabric samples: (**a**) areal density, (**b**) thickness.

Figure 4 shows a parametric comparison of areal density (GSM) and thickness for the samples. The results of areal density shown in Figure 4a indicate that samples with a cross-relief structure (S2, S5, S8, S11 and S14) show relatively higher areal densities. The higher areal density of the cross-relief structure is due to the presence of consecutive miss/float stitches, which pull the loops together. As a result, the fabric areal density/GSM increases [31]. The samples with a vertical tubular structure resulted in a lower areal density/GSM. This is because the vertical tubular fabric is a hollow and open/porous structure. The porosity is inversely related to tightness and thus results in a reduction in areal density/GSM. These results are supported by the reported literature [31,32]. It was observed that samples produced using 50% Nomex and 50% carbon fibers show relatively higher weight/GSM (gram per square meter). The higher GSM of the fabric samples with 50% carbon fibers is due to the relatively higher density of carbon fibers themselves [33]. Relatively lower GSM values were observed in the samples composed of 70% Nomex and 30% carbon fibers. 

Samples with cross-relief structure have relatively higher thickness, as shown in Figure 4b. The cross-relief structure consists of consecutive miss stitches, which make the structure compact, and the packed structure results in increased thickness. Fabrics with higher wale density (wales/cm) result in higher thickness [34]. Lower thickness is observed in the fabric samples with a vertical tubular structure due to its open geometry [35]. Fabric samples composed of 50% carbon and 50% Nomex show relatively higher thickness. The higher content of carbon fibers increases the thickness as they are stiffer and make the structure bulkier. Minimum thickness was observed in the sample composed of 70% Protex and 30% Nomex fibers. This specific sample has minimum hairiness and unevenness, resulting in a finer yarn diameter, which decreases the thickness in the fabric [36]. It is inferred that in most cases, the areal density is directly proportional to the thickness of the fabrics. The areal density and thickness values can be related to the fabric structure. It can be observed that a cross-relief fabric structure results in higher areal density as well as higher thickness, while a vertical tubular fabric structure results in lower areal density and lower thickness. These results are supported by the reported literature [35,36].

### 3.2. Mechanical Properties and Serviceability

#### 3.2.1. Tensile Performance

**Figure 5:**
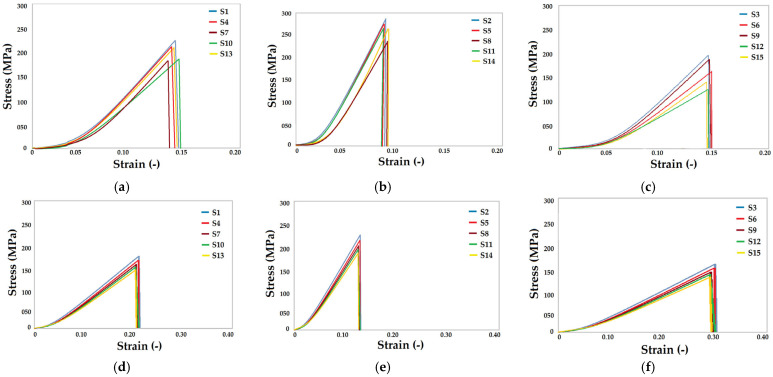
Stress–strain curves for the tensile behavior of fabric samples, (**a**) cross-miss structures in wale direction, (**b**) cross-relief structures in the wale direction, (**c**) vertical tubular structures in the wale direction, (**d**) cross-miss structures in the course direction, (**e**) cross-relief structures in the course direction, (**f**) vertical tubular structures in the course direction.

The tensile test of the fabric samples was carried out in the wale (length wise) and course (width wise) direction. Standard test methods were used to determine the tensile strength, elongation as well as the elastic modulus. Figure 5a–c show the stress–strain curves in the wale direction for the various knitted fabric structures. Similarly, Figure 5d–f show the stress–strain behavior of fabric samples in the course direction. In order to determine the influence of different knitted architectures on the tensile properties, the stress–strain curves of fabric samples with different blend compositions are presented in groups. It can be observed that the cross-relief knitted structure results in the highest tensile strength as well as the elastic modulus as compared to cross-miss and vertical tubular geometry. This was observed in all the blends of fire-resistant fibers. Such observations are supported by the previous literature where cross-relief geometry resulted in a higher tensile strength and modulus [37,38,39]. The compact and robust nature of the cross-relief geometry is responsible for its superior mechanical performance.

Among the different blends of inherently fire-resistant fibers, the 70% Protex/30% Nomex blend showed the maximum tensile strength and modulus. It is very interesting to note that both Nomex and Protex are mechanically stronger fibers used in the current study. However, in a blended yarn used for knitting and weaving purposes, the overall mechanical performance of the fabric is not a direct function of the constituent fiber tenacity or modulus. It also depends on the yarn compactness and porosity, the inter-fiber friction and the fiber length. Taking all these parameters into account, the 70% Protex/30% Nomex blended yarn proved to be stronger than 100% Nomex yarn. Therefore, the mechanical performance of the fabric samples developed from such yarns is superior to the other blends used.

As normally expected, the tensile strength and modulus (based on the slope of the stress–strain curves) was higher in the wale (length) direction, as shown in Figure 5a–c, compared to the course (width) direction shown in Figure 5d–f. This is related to the higher stitch density and inter-loop friction, which restricts the tensile deformation in the longitudinal direction. These results are supported by the reported literature [40,41]. On the other hand, the loop density in the course direction is relatively lower, resulting in a lower tensile strength and modulus.

Furthermore, the effects of different blend compositions are more distinct in the wale (longitudinal) direction. This is due to there being significantly more inter-fiber slippage along the yarn axis. In the course direction, there is a transverse loading on the loops of yarn, and the deformation is mainly due to there being a change in the curvature of the loops rather than the yarn deformation. Owing to the similar loop geometry (height and length), very similar behavior is observed for different blends of fibers in this research. It is noteworthy that a lower amount of load is required to deform a loop in the width direction than to deform the yarn along the axial direction. Therefore, the strain in the course direction is relatively higher than the strain in the wale direction. This holds good for all the fiber blends and all the different knitted architecture used. These results are supported by the reported literature [42,43].

#### 3.2.2. Bursting Performance

**Figure 6:**
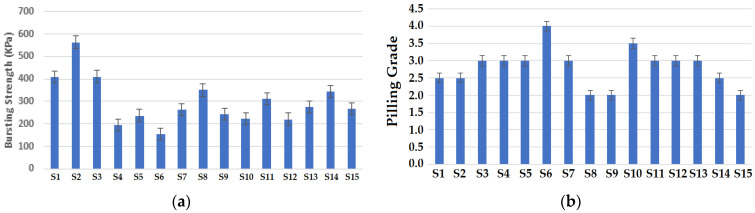
Results of (**a**) bursting strength, (**b**) pilling grade.

The results of bursting strength are shown in Figure 6a. It is depicted that samples with a cross-relief structure exhibited the highest bursting strength followed by the other two structures. The cross-relief structure consists of a higher amount of miss stitches as compared to other structures. The presence of consecutive miss stitches produces a compact structure, as reported in the literature [44]. The vertical tubular structure showed the minimum bursting strength due to presence of tuck stitches. The presence of tuck stitches in the structure of the knitted fabric decreases the bursting strength while increasing the porosity of the fabrics. These results are supported by the reported literature [45]. Vertical tubular structures also showed minimum thickness and GSM values which have an impact on the bursting strength of the fabrics. It was observed that fabric samples composed of 70% Protex/30% Nomex fibers showed the highest bursting strength. This can be attributed to the highest elongation and single yarn strength of the 70% Protex and 30% Nomex fiber blended yarn used to develop the samples. This is also supported by the reported literature [45]. Lower values of bursting strength were observed in the samples composed of 50% Nomex and 50% carbon fibers. This can be due to the relatively lower tenacity of carbon fibers as reported in previous studies [40,41,42,43,44,45].

#### 3.2.3. Pilling Resistance

The results of pilling resistance test are shown in Figure 6b. It was observed that the vertical tubular structure shows the highest pilling resistance. It can be due to the influence of the tubular construction. The tubular structure with tuck stitches introduces pores in the structure, and such pores ultimately increase the pilling resistance. The findings are supported by the reported literature [46]. The results also suggest that the sample with a cross-miss structure shows relatively higher resistance to pilling. The cross-miss structure is a balanced structure composed of alternate miss and knit stitches. Its balanced and compact structure results in relatively higher pilling resistance as per the literature [47]. Minimum pilling resistance was observed in the samples made with the cross-relief structure. This structure consists of knit and miss stitches at different locations, which forms a kind of motif in the fabric. The motif design pattern in the cross-miss structure is subjected to higher frictional forces and as a result, there is higher occurrence of pills. Similar results were also reported previously [48].

Pilling is a process of accumulating the broken fibers which protrude from the fabric surface. High pilling is an indication of the weaker mechanical performance of fibers and the generation of static charge due to abrasion. The broken fibers create ball-like structures, which stick to the surface of the abraded fabric. This usually happens during the practical usage of the garments. A higher number of pills on the surface also increases the flammability of the fabric. Therefore, the flammability and pilling resistance can be studied together [47,48].

**Figure 7:**
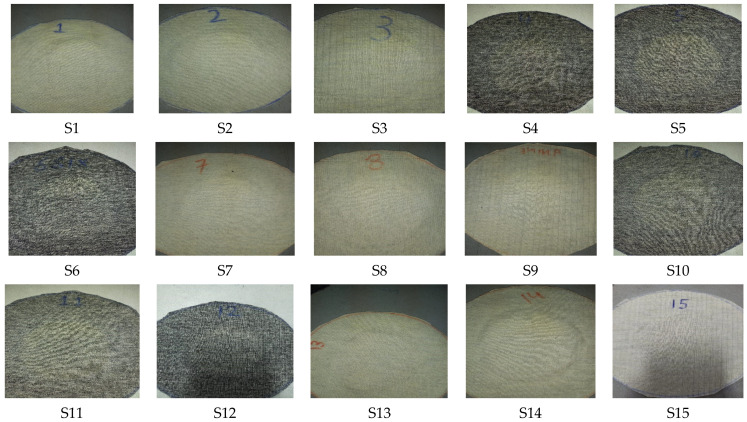
Photographic images of fabric samples after pilling test (abrasion for 18,000 cycles).

It is also noteworthy that the blend of 70% Protex and 30% Nomex fibers showed the lowest pilling resistance. This is due to the inherent characteristics of modacrylic (Protex) fibers having the tendency to pill easily. A higher pilling resistance was shown by the samples composed of 50% Nomex/50% carbon fibers. This can be attributed to the brittle nature of both carbon and Nomex fibers, which enhances the pilling resistance. The photographic images of the fabric samples after the pilling test (abrasion for 18,000 cycles) are shown in Figure 7.

### 3.3. Thermo-Physiological Comfort Properties

Thermo-physiological comfort is a very important aspect of fabric materials used in next-to-skin applications [49]. This includes the air permeability, thermal resistance and overall moisture management capacity (OMMC).

#### 3.3.1. Air Permeability

**Figure 8:**
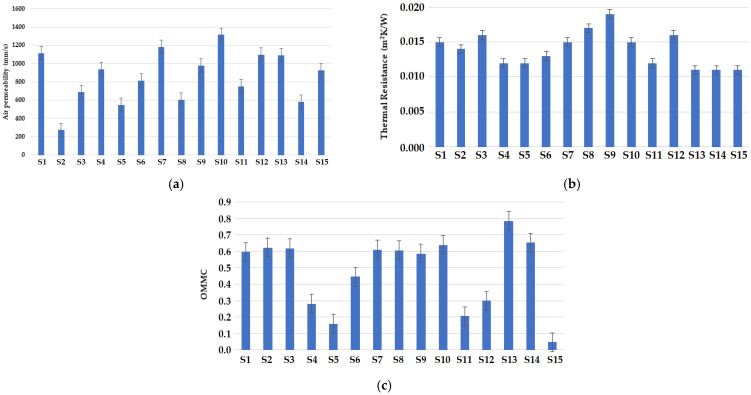
Thermo-physiological comfort properties of the samples: (**a**) air permeability, (**b**) thermal resistance, and (**c**) overall moisture management capacity (OMMC).

The air permeability measurement results for the fabric samples are shown in Figure 8a. It was observed that the samples with a cross-miss structure show relatively higher air permeability. It depends upon the thickness, porosity, or tightness factor, as identified by researchers [45]. The cross-miss structure consists of alternate miss and knit stitches, which increase the porosity of the fabric. The higher air permeability value of the sample can be attributed to the lower weight/GSM and lower number of loops (wales or courses/cm). It has been concluded by the previous researchers that fabrics with a lower number of loops/cm show higher air permeability [46]. The sample with a cross-relief structure shows the lowest air permeability due to higher GSM as compared to other samples. The samples with higher GSM are usually denser with lower porosity. It is obvious that the cross-relief structure consists of a higher amount of miss stitches. These consecutive float stitches make the fabric more compact and less permeable for air. Such inferences are based on the reported literature [47,48].

It was observed that the fiber blend composition does not have any significant effect on the air permeability of the fabric samples developed. The highest air permeability was observed in the sample composed of 70% Nomex and 30% carbon fibers. The lowest air permeability was observed in the sample with 50% Nomex and 50% carbon fibers due to the presence of high-density carbon fibers, which tend to increase the packing of yarn and decrease the porosity. Such findings are supported by the literature [49].

#### 3.3.2. Thermal Resistance

The thermal resistance of the fabric samples is shown in Figure 8b. It can be observed that the highest thermal resistance was obtained for the sample with a vertical tubular structure. The vertical tubular structure consists of dead air pockets which can entrap stagnant air as an insulating medium and increases the thermal resistance. The cross-relief structure shows relatively higher thickness and thus higher thermal resistance. Thermal resistance has a direct relationship with the thickness of the fabric samples as per the literature [49,50,51]. A blend of 70% Protex and 30% Nomex fibers developed with a vertical tubular knitting structure showed the maximum thermal resistance. Protex is a kind of modacrylic fiber, which can be easily blended with other constituent fibers and tends to enhance the overall thermal resistance of the blended fabric [51].

It can also be observed from Figure 8b that fabric samples with carbon fibers as a constituent material show higher thermal resistance. Polyacrylonitrile (PAN)-based carbon fibers have lower thermal conductivity [52]. This can also be associated with the hairiness in the yarn containing carbon fibers as mentioned earlier. A higher amount of hairiness in the yarn increases the microporosity of the fabric and the thermal resistance [49,50,51].

It was observed that samples with 70% Nomex and 30% viscose fibers showed the lowest thermal resistance due to the relatively lower thermal resistance of viscose fibers [53].

#### 3.3.3. Overall Moisture Management Capacity (OMMC)

The overall moisture management capacity (OMMC) of the fabric samples is shown in Figure 8c. The moisture absorbance or transmission depends on the microporosity of the knitted fabric structure. The microporosity is defined as the micro spaces between the fibers present in the yarn. This promotes capillary action and governs the liquid moisture transportation in the fabric. Moreover, the fabric construction is responsible for liquid water transportation because of the inter-yarn spaces [54]. With respect to the fabric structure, it was observed that the cross-miss structure showed the maximum OMMC due to alternate miss and knit stitches in the diagonal direction. This arrangement formed inter-yarn micro-spaces and capillaries, which lead to better moisture transportation. The findings are in accordance with the previous literature [54,55].

Thinner fabric materials with relatively lower areal density (GSM) show higher moisture diffusion, which leads to better moisture management. The water vapor evaporation rate decreases with the increase in the thickness of fabrics. It was also observed that the cross-relief structure showed the lowest OMMC. It was also observed by several other researchers [56,57,58].

It was observed that the fabric samples composed of 100% Nomex fibers and 70% Nomex + 30% viscose fibers show better moisture management properties, which can be attributed to the inherent characteristics of Nomex fibers. It has been concluded by researchers that Nomex fibers have relatively better wicking properties, which tend to enhance the overall moisture management [59]. The higher OMMC values for 70% Nomex + 30% viscose fibers may be due to the presence of hygroscopic viscose fibers. Researchers mentioned that viscose fibers blended with other fibers help enhance absorbency-related properties [59,60]. It was also observed that the sample composed of 70% Protex + 30% Nomex fibers shows relatively superior moisture management properties. Protex is a modacrylic fiber which has been reported to exhibit superior moisture management performance [61].

The results show that the sample composed of 50/50 carbon/Nomex displayed the lowest OMMC values due to the hydrophobic nature of the carbon fiber, which is resistant to moisture absorption [62]. The carbon fibers used in the blends show very poor moisture management-related properties (e.g., moisture regain and moisture content), as shown in Table 1. It was also noted that the OMMC values tend to decrease as the content of carbon fibers increases in the blend. Similar findings are reported by several researchers [63,64].

### 3.4. Tactile/Touching Comfort Properties

**Figure 9:**
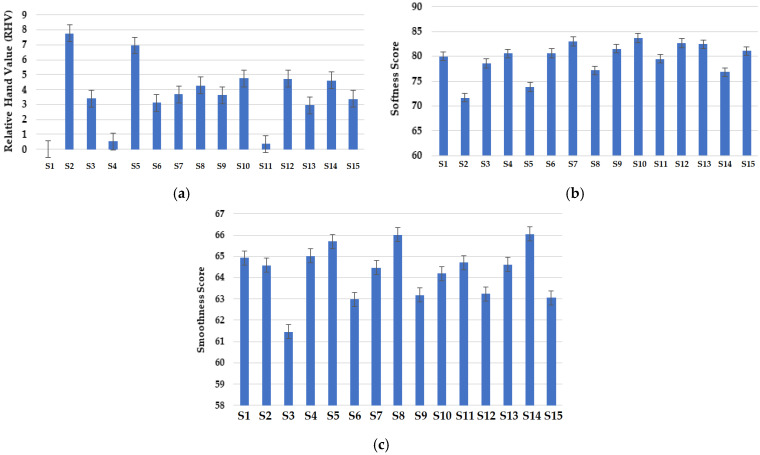
Tactile comfort properties of samples; (**a**) relative hand value (RHV), (**b**) softness score, (**c**) smoothness score.

The tactile comfort properties of the knitted fabric samples are shown in Figure 9. The relative hand values (RHVs) of the samples are shown in Figure 9a. The sample composed of 100% Nomex fibers and knitted with a vertical tubular structure was treated as the reference sample. A lower RHV means closer to the reference sample, and a higher RHV means that the concerned sample is far from the reference sample. The results show that the sample composed of 50% Nomex + 50% carbon fibers with a vertical tubular structure exhibited the lowest RHV (closest to the reference sample). This can be attributed to the blend composition including Nomex fibers. It was also noted that samples composed of 70% Protex + 30% Nomex and 70% Nomex + 30% viscose fibers showed relatively lower RHVs than the other samples. The Protex fiber also shows an inherently soft handle/touch. It was noted that the fabric structure has a dominant effect on the RHV. Samples with vertical tubular structures exhibited relatively lower RHVs. Similar structural characteristics resulted in similar tactile properties. The sample composed of 100% Nomex fibers with a cross-relief knitting structure displayed the highest RHV. Indeed, all the samples with the cross-relief structure exhibited relatively higher RHVs. The findings are supported by the previous literature [64,65,66].

The softness scores of the fabric samples were obtained from the Phabrometer-3 fabric evaluation system and are presented in Figure 9b. It can be observed that the sample composed of 70% Nomex + 30% carbon fibers showed the highest softness score. This can be related to the relatively lower density of Nomex fibers. It has been concluded by researchers that a lower fiber density results in softer surface handle/touch-related properties [65]. It was noted that the sample with a cross-miss structure displayed a maximum softness value. It was also found that fabric density and compression properties are related to fabric softness [65,66]. The higher density of a cross-miss structure resulted in higher softness values [66]. The minimum softness value was shown by the sample composed of 50% Nomex + 50% carbon fibers. This is because of the brittle nature of carbon fibers. Samples with similar blend compositions and a cross-relief structure showed relatively lower softness values. The samples composed of 70% Protex fibers also resulted in relatively higher softness values due to the inherent softness of modacrylic fibers.

The smoothness scores of the samples are shown in Figure 9c. It was observed that the samples composed of 70% Nomex + 30% viscose fibers resulted in higher smoothness values. Viscose fibers tend to increase the smoothness of the fabric surface. It was revealed that the fabric sample with a cross-relief structure showed the maximum smoothness. A minimum value of fabric smoothness was observed in the sample composed of 50% Nomex + 50% carbon fibers due to the brittle nature of both carbon and Nomex fibers [67]. It was also found that the sample with a vertical tubular structure showed minimum smoothness because of the tube-like structure of the fabric.

Based on the results, the softness among the different knitted structures was ranked in the following order: cross-miss > vertical tubular > cross-relief. The smoothness was ranked in the following order: cross-relief > cross-miss > vertical tubular. These results are in accordance with the reported literature [60,61,62,63,64,65].

### 3.5. Dimensional Stability

**Figure 10:**
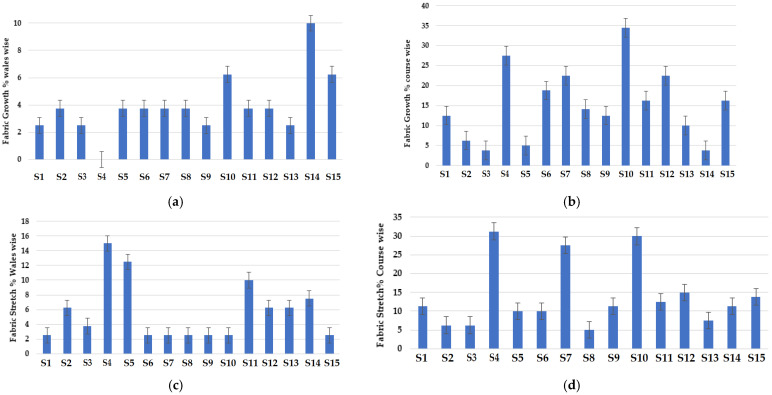
Dimensional stability of knitted fabric samples: (**a**) fabric growth % (wale wise), (**b**) fabric growth % (course wise), (**c**) fabric stretch % (wale wise), (**d**) fabric stretch % (course wise).

The dimensional stability-related characteristics of the knitted fabric samples are shown in Figure 10. The fabric growth (i.e., residual extension after removal of load) in the wale (length wise) direction is shown in Figure 10a. Samples containing 70% Nomex + 30% viscose fibers showed maximum growth in the wale direction. The viscose fibers show relatively higher elongation according to the reported literature [68]. It was observed that the presence of a higher percentage of Nomex fibers increased the fabric growth. Nomex fibers are composed of oriented meta phenylene groups, which result in the bending of the polymer chains and reduce rigidity. This ultimately increases the fabric growth. It was obvious from the results that lower fabric growth values were observed in the walewise direction in the samples containing carbon due to the much lower elongation. This is attributed to the relatively higher crystallinity of carbon fibers. The cross-miss knitted structure also influences the dimensional stability. The structure consists of alternate miss and knit stitches. It is well known that the yarns bent in the form of a loop result in higher stretch and recovery [60,61,62]. As a result, the fabric material exhibited minimum growth (i.e., residual extension after removal of load).

From Figure 10b, it can be observed that fabric growth in the course (width-wise) direction is the highest for the sample composed of 70% Nomex + 30% carbon fibers. The sample knitted with a cross-miss structure showed the maximum growth value in the course direction. The knitted fabric sample with a lower weight/GSM and density tends to show lower growth [61,62,63,64]. It is also obvious in the weft knitted structures that fabrics that have higher wales/cm show greater stability in the width-wise direction. Similarly, fabrics with higher courses/cm show better stability in the length direction [65,66,67]. It was also observed that the lowest value of fabric growth in the course direction was observed in the samples with a 70/30 blend of Nomex/viscose fibers. It was observed that the sample knitted with a cross-relief structure showed minimum growth values due to there being a higher amount of miss stitches. Knitted fabrics having float stitches tend to pull the adjacent loops and improve the fabric stability [68,69,70,71,72]. The floating yarn becomes vertically straight in the course (width) direction and tends to resist the fabric growth. The structure of the knitted fabric was found to be the dominant factor in determining the fabric growth [73,74].

It was observed that the cross-relief structure showed the highest growth in the wale direction due to a higher number of wales/cm. It was also observed that the cross-miss structure showed the lowest fabric growth in the wale direction and maximum growth in the course direction due to the presence of float stitches. The results are supported by the literature [75].

Figure 10c shows the stretch % in the wale direction for the different knitted fabric samples. The sample composed of 70% Protex + 30% Nomex fibers resulted in the lowest stretch along the wale direction due to there being a higher number of courses and wales/cm. The fabric samples with a higher number of course and wales/cm show better dimensional stability. This results in higher stretch and recovery, as concluded by several researchers [76,77]. The fabric sample composed of 50% Nomex + 50% carbon fibers exhibited maximum fabric stretch in the wale direction. This can be attributed to the inherently lower extension of the carbon fibers. The samples knitted with a cross-miss structure exhibited minimum fabric stretch due to the float stitches [77].

Samples composed of 50% Nomex + 50% carbon fibers displayed a relatively higher stretch in the course-wise direction, as shown in Figure 10d. It also became evident from the results that samples with a similar fiber blend composition and cross-miss structure showed the maximum stretch. As already discussed, the fabrics with high course and wale density result in better stretch and recovery performance [78,79,80]. Samples composed of 70% Protex + 30% Nomex fibers showed minimum stretch in the course direction due to the relatively higher tenacity of Protex fibers. The dimensional stability-related properties of knitted fabrics, e.g., growth (residual extension), stretch and recovery, mainly depend on the construction parameters. It was observed that the cross-relief structure showed the maximum stretch in the wale direction. On the other hand, the cross-miss structure showed maximum stretch and minimum recovery, indicating a relatively poor dimensional stability [78,79,80].

### 3.6. Flame Resistance

#### 3.6.1. Vertical Flammability Test

**Figure 11:**
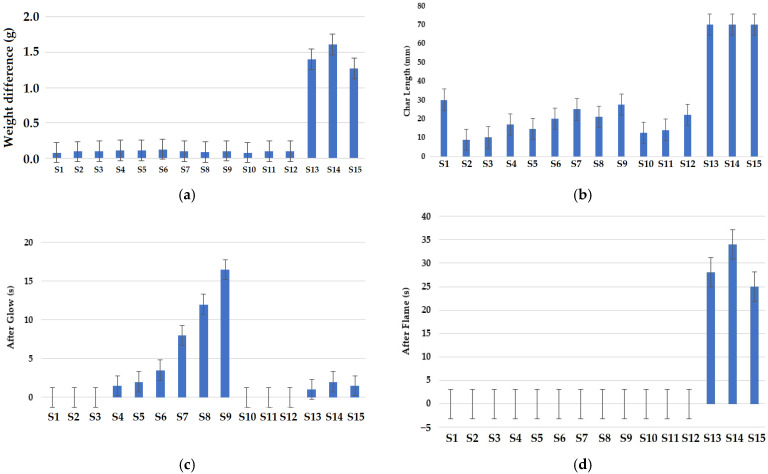
Results of vertical flammability test of knitted fabric samples; (**a**) weight difference, (**b**) char length, (**c**) after glow time, (**d**) after flame time.

The results of the vertical flammability test are shown in Figure 11. It was observed that the sample composed of 70% Nomex + 30% viscose fibers was fully burnt. This can be due to the presence of viscose fibers with relatively lower flame resistance [50]. It can be observed from Figure 11a that the sample composed of 70% Nomex + 30% viscose fibers shows maximum weight loss. This confirms the reported views that viscose fibers have much lower flame resistance and a low limiting oxygen index (LOI) value of about 18.9% due to which they burn easily [51]. Apart from the fabrics that underwent complete burning, observation regarding the weight loss suggested that samples composed of 100% Nomex and 70% Nomex + 30% carbon fibers resulted in much lower weight loss. This can be attributed to the inherent fire-retardance properties of meta-aramid (Nomex) and carbon fibers. The presence of carbon fibers in a blend enhances the fire resistance of fabrics [52]. Sometimes, a minor weight loss can be observed due to the removal of moisture. Carbon fibers have the lowest moisture content, and therefore, no significant weight loss was observed. It was found that the fabric structure does not have a significant effect on the weight loss after burning. The error bars in the figures indicate the upper and lower limits for the corresponding samples.

According to the Standard on Emergency Services Work Apparel of the National Fire Protection Association, Massachusetts, USA (NFPA 1975), it is recommended that the char length should not be more than 15 cm [81]. The results shown in Figure 11b depict the char length obtained in the samples. The results reveal that the fabric sample composed of 100% Nomex fibers showed minimum char length due to the inherent flame-resistant characteristic of meta-aramid fibers containing benzene rings. It was observed that the fabric sample composed of 70% Nomex + 30% carbon fibers also exhibited much lower char length. As mentioned earlier, the higher resistance to fire in case of carbon and Nomex fibers is responsible for lower char lengths. Both types of fibers are non-combustible, and specifically, carbon fibers exhibit a very high LOI (almost >40) [74]. It is noteworthy that among the different knitted fabric structures, the cross-relief structure showed minimum char length. This could be because of the tight and compact structure, which prevents the formation and propagation of combustible gases and thus minimizes burning. This ultimately resulted in a minimal char length.

The afterglow time for different fabric samples is shown in Figure 11c. It can be observed that the sample composed of 70% Nomex + 30% carbon fibers showed the best results. In this sample, zero afterglow time was observed, while all other samples show afterglow for some time. Therefore, it can be inferred that the combination of Nomex and carbon fibers shows better results in terms of self-extinguishing of the blended fabric [53].

The after-flame time for the fabric samples is shown in Figure 11d. It can be observed that there was zero after-flame time in all the samples except for the sample composed of 70% Nomex + 30% viscose fibers, which was completely burnt. It was noted that samples knitted with a cross-relief structure showed maximum after-flame time. Maximum smoke was produced during the burning of samples composed of 70% Nomex + 30% viscose fibers, which was graded with the highest after-flame time. Furthermore, the sample composed of 70:30 Protex:Nomex fibers showed a fair amount of smoke. Very little smoke was observed while burning 50:50 carbon:Nomex fiber-based materials. During the burning of samples composed of 100% Nomex and 70% Nomex + 30% carbon fibers, no smoke was produced. The burning behavior is mainly affected by the fiber content and their chemical composition. The carbon fibers showed much better flame resistance than the other types of fibers. It is due to the heat treatment during their production from polyacrylonitrile (PAN) precursors at a very high temperature (600–1100 °C) [54].

**Figure 12:**
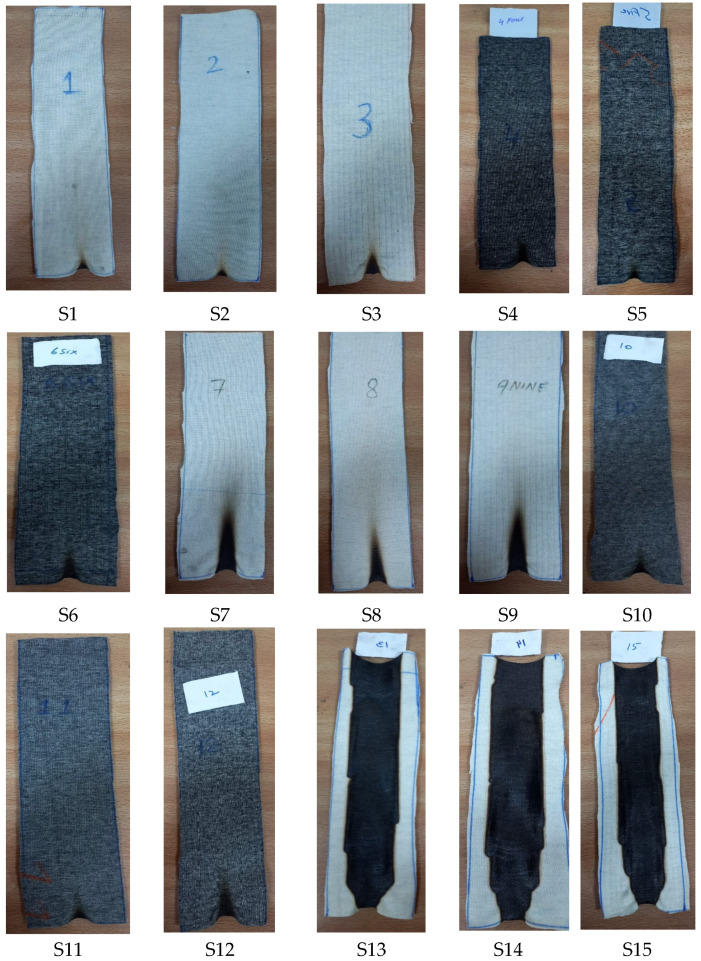
Photographic images of the samples after vertical flammability test.

The photographic images of the knitted fabric samples after the vertical flammability test are shown in Figure 12.

#### 3.6.2. Thermogravimetric Analysis (TGA)

**Figure 13:**
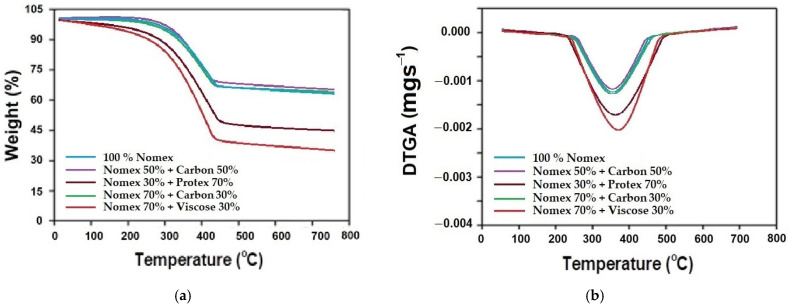
Thermogravimetric analysis under oxidative conditions for the samples with different fiber blend compositions: (**a**) TGA, (**b**) DTGA.

Thermogravimetric analysis (TGA) was performed under oxidative conditions for the different samples to analyze the effect of blend composition on thermal stability. The change/loss in mass was measured as a function of rise in temperature. All the samples were subjected to temperatures ranging from 25 to 700 °C at a heating rate of 10 °C/min. The samples behaved differently, and variable mass degradation was observed. The TGA curves for the samples are shown in Figure 13a. Differential thermogravimetric analysis (DTGA) curves of samples are also shown in Figure 13b.

From Figure 13a, it can be observed that the 100% Nomex, 50% Nomex+ 50% carbon, and the 70% Nomex +30% carbon fiber-based samples show minimum mass loss under oxidation and combustion. Up to around 300 °C, the mass loss was almost negligible. Subsequently, there was about 28% mass loss from 300 to 400 °C. This loss can be due to the pyrolysis of the fiber surface under oxidation and burning of char. However, beyond 400 °C, not much mass loss was observed. The blend of 30% Nomex + 70% Protex fibers showed about 55% mass loss. The sample containing 70% Nomex + 30% viscose fibers showed a maximum mass loss of about 58%. The modacrylic and viscose fibers produced more combustible gases, and char as was observed in vertical flammability tests. The results of TGA showed similar trends to vertical flammability. Such results are also validated by the reported literature [74,75,76,77].

The derivative results of TGA under oxidative conditions were also plotted to obtain a clearer picture of the events that occurred during combustion. The ignition points in the TGA curve (with higher weight loss %) were indicated by the peak on the DTGA curves shown in Figure 13b. The DTGA curves clearly indicate the existence of only one main mass-loss region, which is always located between 300 and 400 °C. This region can be attributed to the thermal decomposition of the fibers under oxidative conditions. Several other studies have reported findings that are similar to the present research [70,72,73]. The results indicated that samples of 100% Nomex and blends of Nomex + carbon underwent minimal oxidative and combustion-related decomposition, whereas blends of Nomex + Protex and Nomex + viscose resulted in relatively higher weight loss due to the oxidative decomposition of modacrylic or viscose fibers in the blend. The findings showed a similar trend, as was observed in vertical flammability tests. The fabric structure had no significant influence on the thermal degradation behavior at extremely high temperatures.

#### 3.6.3. Cone Calorimetry

The cone calorimeter was used to evaluate the ignition time, t_ig_ (s), peak heat release rate, PHRR (kW/m^2^), total oxygen consumed, TOC (g), average mass loss rate, Av MLR (g/s), and average CO_2_ yield, Av CO_2_ (kg/kg). Table 5 summarizes the identified calorimetric parameters for the different blend compositions investigated.

**Figure 14:**
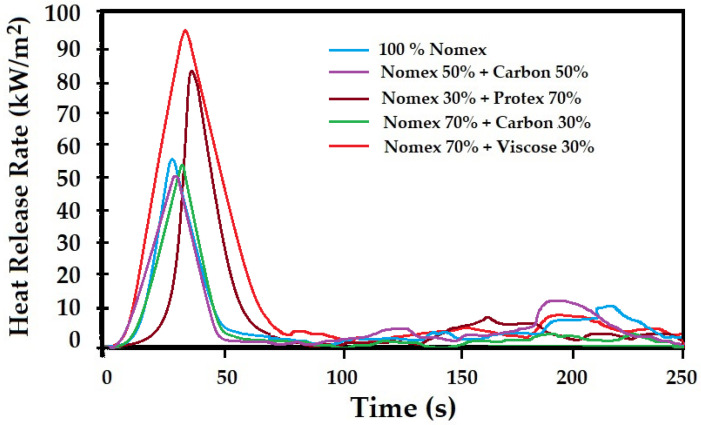
The peak heat release rate (PHRR) for the samples with different fiber compositions.

The peak heat release rate (PHRR) is the major parameter to evaluate the flame resistance of materials. Figure 14 shows the heat release rates of different blend compositions in the knitted fabrics. The heat release rate (HRR) curves show a distinct sharp peak followed by a drop in HRR. Subsequently, there was a slight rise in HRR spread over a period of time. The initial peak in HRR may be attributed to the surface pyrolysis, and the drop is related to the char formation. The subsequent small rise may be attributed to the burning of the unburnt substrate and an increase in the bulk temperature of the fiber material. The significant difference in behavior can be observed for different fiber blends.

It can be observed from Figure 14 that the minimum heat release rate was obtained for the 50% Nomex + 50% carbon fiber blend. The 100% Nomex and 70% Nomex + 30% carbon fiber blend also exhibited a similar peak heat release rate. It is therefore evident and established that Nomex and carbon fiber blends are the most robust compositions for flame protection materials. These results are similar to the observations of vertical flammability as well as TGA. Such findings were also reported in the literature [82,83,84]. The blends of Nomex + Protex and Nomex + viscose showed a much higher heat release rate, which is attributed to the decomposition and burning of modacrylic and viscose fibers, respectively.

The ignition time was also observed to be higher for the 100% Nomex and Nomex + carbon fiber blends. The Nomex + Protex and Nomex + viscose fiber blends showed much lower ignition time. These blends with modacrylic as well as cellulosic viscose fibers resulted in higher oxygen consumption and CO_2_ yield. These are indications of relatively lower flame protection with Protex and viscose fiber blends along with Nomex fibers. The mass loss was further noted to be slightly higher for these blend compositions. The results were similar to trends observed in TGA and DTGA curves. The reported literature also validated the findings of this work [82,83,84,85].

### 3.7. Radiant Heat Resistance

**Figure 15:**
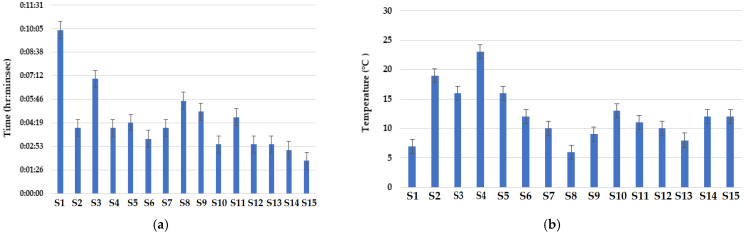
Results of radiant heat resistance: (**a**) radiant heat time interval, (**b**) radiant heat temperature gradient.

The results of radiant heat resistance are shown in Figure 15a,b. The fabric material composed of 70% Protex + 30% Nomex fibers showed relatively good results. In these samples, a relatively lower temperature rise was observed for a longer time duration. The sample knitted with a cross-relief structure exhibited a minimum temperature rise, which may be due to the consecutive miss stitches present in a greater amount, making the structure more compact. The fabric sample composed of 70% Protex + 30% Nomex fibers knitted with a cross-relief structure was found to be the best when compared with the control sample composed of 100% Nomex fibers. Protex fibers showed better resistance to radiant heat, as observed by researchers [55]. This can be attributed to the relatively denser construction of a cross-relief structure and the inherently fire-resistant characteristics of Protex fibers [31]. The control sample did not show good results in terms of radiant heat resistance.

The sample composed of 50% Nomex + 50% carbon fibers showed the lowest radiant heat resistance. Furthermore, the sample knitted with the cross-miss structure showed the lowest resistance against radiant heat. Although the Nomex fibers and carbon fibers have higher resistance to radiant heat [20], in this case, the fabric construction/architecture was the dominant factor. The cross-miss structure contains a relatively higher number of knit stitches as compared to other structures. The alternate miss and knit stitches allow the heat to pass through the fabric. The cross-miss structure has bigger pores and thus showed a relatively lower resistance to heat transfer [56]. This sample allows maximum radiant heat to pass through, resulting in a maximum increase in temperature.

### 3.8. Convective Heat Resistance

**Figure 16:**
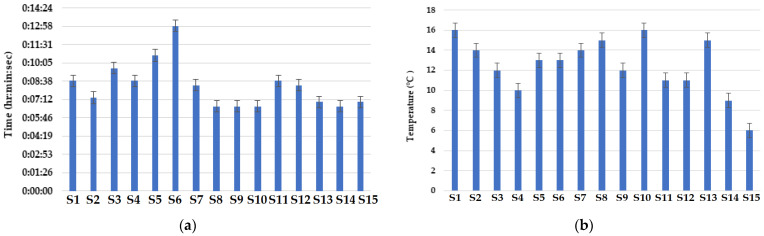
Convective heat resistance of the knitted fabric samples: (a) time interval, (b) temperature gradient.

The results of convective heat resistance of the knitted fabric samples are shown in Figure 16a,b. It can be observed that the 70% Nomex + 30% viscose fiber blended fabric sample showed the best results due to the inherent fire-resistant properties of Nomex fibers. The amide linkages with aromatic rings present in aramid fibers like Nomex result in a high fire resistivity. The fabric sample knitted with the vertical tubular structure showed the maximum convective heat resistance. This sample also showed the minimum rise in temperature. In the vertical tubular structure, there are dead air pockets which behave as insulators to resist the loss of heat across the fabric sample. The 30% Nomex + 70% Protex fiber blended material showed very poor resistance to convective heat transfer. Convective heat transfer depends upon the microstructure of fibers. The modacrylic fibers performed poorly against convective heat due to their surface geometry [57]. The poor results shown by the Protex fibers may be because of their soft nature and damage to the fiber. Such materials exhibited a maximum rise in temperature among all the knitted fabric samples. The sample with a relief knitted structure showed the highest rise in temperature. This temperature rise can be attributed to the long floating stitches which tend to make the fabric thinner. The thinner fabrics enable easier heat transfer and offer minimum resistance. These results are similar to the findings of several other researchers [54,55,56,57,58].

The time required to raise the temperature is an important factor during convective heat transfer. The results of convective heat resistance revealed that the sample composed of 50% Nomex +50% carbon fibers showed the best results based on maximum time interval. These samples underwent the temperature rise involving the maximum time interval. The aramid fibers have inherently high flame resistance due to meta-aramid linkages and their non-crystalline nature [60,62]. Carbon fibers also exhibit high resistance to heat due to their inherent FR character [61]. Therefore, the sample showed very high resistance against the convective heat. It is well known that fabrics with higher thickness decrease the amount of heat transfer through the structure and enhance the fire and heat protection performance [62]. It has been confirmed by several researchers that higher thickness and weight/GSM decrease the thermal conductivity, which ultimately increases the thermal resistance [60,61,62,63].

## 4. Conclusions

The firefighters’ clothing material is required to shield the wearers from all possible hazards during their work. In this study, different blends of inherently flame-resistant fibers, i.e., Nomex, Protex, carbon and viscose, were used to produce derivatives of interlock knitted fabrics. Three different derivative structures were used, e.g., miss, relief and vertical tubular.

The fabric material composed of 70% Protex + 30% Nomex fibers knitted with a relief structure showed the highest tensile and bursting strength, while the miss and vertical tubular structures showed the highest pilling resistance. It was found that 70% Protex + 30% Nomex fiber blends showed the best performance in terms of dimensional stability. The sample with a vertical tubular structure showed excellent dimensional stability in the wale direction (length wise), and the relief structure showed the best dimensional stability in the course direction (width wise).

It was found that the composition of 70% Protex + 30% Nomex fibers exhibited the highest thermal resistance, best moisture management capacity and moderate air permeability. Among the different knitted structures, vertical tubular fabrics exhibited the best thermo-physiological performance. It was found that the blend compositions of 70% Protex + 30% Nomex and 70% Nomex + 30% carbon fibers showed the best tactile comfort in terms of relative hand value (RHV), softness and smoothness. The fabric sample knitted with a relief structure showed the best tactile comfort properties due to the presence of a higher number of tuck stitches.

It was found that the blend of Nomex and carbon fibers showed the best flame resistance performance having a minor char length, minimum weight loss and no after-glow time. These findings were confirmed with results from TGA and DTGA under oxidative conditions and by cone calorimetry. The heat release rate (HRR) was minimum for the Nomex and carbon fiber blends. The fabric sample with a relief structure showed the maximum flame resistance. It was found that the fabric sample composed of 70% Protex + 30% Nomex fibers showed the best radiant heat resistance. The sample with a relief structure showed the highest radiant heat resistance due to the presence of tuck stitches and a compact structure. It was found that the 50% Nomex + 50% carbon fiber blends showed the best convective heat resistance owing to their inherent fiber characteristics. The vertical tubular structure was the best for convective heat resistance among the derivatives of interlock knit structures.

The findings of this research can be used to engineer flame-resistant fabric materials with optimum mechanical performance, dimensional stability, fire protection and comfort properties in the practical field. In the future, the significance of each contributing factor can be investigated by statistical analysis, and all responses can be optimized.

## Figures and Tables

**Table 1:** Properties of fibers.

Sr. No	Fiber Type	Denier (g/9000 m)	Tenacity (g/denier)	Density (g/cm^3^)	Extension (%)	Moisture Content (%)	Moisture Regain (%)	Length (mm)
1	Nomex (N)	1.63	7.56 ± 0.20	1.37	30.90 ± 1.00	4.70 ± 0.10	4.90 ± 0.10	50 ± 4
2	Protex (P)	1.53	3.48 ± 0.20	1.35	21.80 ± 1.00	0.50 ± 0.04	0.50 ± 0.02	38 ± 3
3	Carbon (C)	2.26	1.57 ± 0.20	1.75–2.00	10.97 ± 1.00	2.30 ± 0.10	2.30 ± 0.10	38 ± 3
4	Viscose (V)	2.02	2.19 ± 0.20	1.53	22.4 7 ± 1.00	7.90 ± 0.10	8.60 ± 0.10	40 ± 4

**Table 2:** Factors and the levels for preparing yarn samples.

Factors	Levels				
	1	2	3	4	5
Fiber Blend % (A)	100% Nomex	Nomex 50%: carbon 50%	Nomex 30%: Protex 70%	Nomex 70%: carbon 30%	Nomex 70%: viscose 30%
Knit Fabric Structure (B)	Cross-relief	Cross-miss	Vertical tubular	-	-

**Table 3:** Designs of knitted fabric samples.

Knitted Structure	Cam Repeat										Fabric View	Knit, Miss, Tuck
Interlock cross-miss	K = knit, T = tuck, M = miss										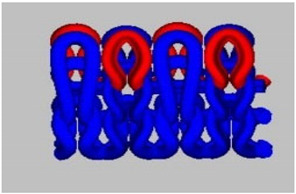	42, 58, 0
	Feeder				F1	F2	F3	F4	F5	F6		
				B	K	M	M	M	K	M		
	dial			A	M	K	M	K	M	M		
				A	K	M	K	M	K	M		
	cylinder			B	M	K	M	K	M	K		
Interlock cross-relief	K = knit, T = tuck, M = miss										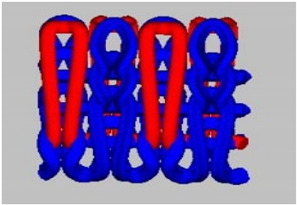	31, 69, 0
	Feeder		F1	F2	F3	F4	F5	F6	F7	F8		
		B	K	M	K	M	M	M	M	M		
	dial	A	M	K	M	K	M	M	M	M		
		A	M	K	M	M	M	K	M	K		
	cylinder	B	K	M	M	M	K	M	K	M		
Vertical tubular	K = knit, T = tuck, M = miss										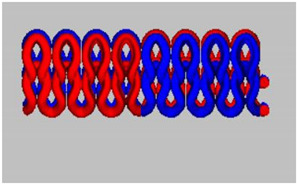	42, 50, 8
	Feeder				F1	F2	F3	F4	F5	F6		
				B	K	M	K	M	K	M		
	dial			A	M	K	T	M	K	M		
				A	K	M	M	K	M	T		
	cylinder			B	M	K	M	M	K	K		

**Table 4:** Design of experiment (DOE).

Sample ID	Fiber Blend	Knitting Structure
S1	100% N	Cross-miss
S2	100% N	Cross-relief
S3	100% N	Vertical tubular
S4	50N/50C	Cross-miss
S5	50N/50C	Cross-relief
S6	50N/50C	Vertical tubular
S7	70P/30N	Cross-miss
S8	70P/30N	Cross-relief
S9	70P/30N	Vertical tubular
S10	70N/30C	Cross-miss
S11	70N/30C	Cross-relief
S12	70N/30C	Vertical tubular
S13	70N/30V	Cross-miss
S14	70N/30V	Cross-relief
S15	70N/30V	Vertical tubular

**Table 5:** Cone calorimeter test results.

Fiber Blend Composition	Ignition Time, t_ig_ (s)	Peak Heat Release Rate, PHRR (kW/m^2^)	Total Oxygen Consumed, TOC (g)	Average Mass Loss Rate, Av MLR (g/s)	Average CO_2_ Yield, Av CO_2_ (kg/kg)
100% Nomex	21 ± 1	55 ± 1	18 ± 1	0.022 ± 0.001	1.92 ± 0.01
Nomex 50%: carbon 50%	25 ± 1	50 ± 1	16 ± 1	0.018 ± 0.001	1.45 ± 0.01
Nomex 30%: Protex 70%	18 ± 1	84 ± 1	20 ± 1	0.025 ± 0.001	2.21 ± 0.01
Nomex 70%: carbon 30%	24 ± 1	53 ± 1	17 ± 1	0.021 ± 0.001	1.62 ± 0.01
Nomex 70%: viscose 30%	15 ± 1	95 ± 1	21 ± 1	0.028 ± 0.001	2.42 ± 0.01

## Data Availability

Data are contained within the article.
